# Molecular Quantum Similarity, Chemical Reactivity and Database Screening of 3D Pharmacophores of the Protein Kinases A, B and G from *Mycobacterium tuberculosis*

**DOI:** 10.3390/molecules22061027

**Published:** 2017-06-21

**Authors:** Alejandro Morales-Bayuelo

**Affiliations:** Fondo Nacional de Desarrollo Científico y Tecnológico (FONDECYT), Proyecto Postdoctoral No. 3150035, Talca 3660300, Chile; alejandr.morales@uandresbello.edu

**Keywords:** tuberculosis, protein kinases A, B, G, 3D pharmacophores, molecular quantum similarity indices, chemical reactivity indices, database screening

## Abstract

*Mycobacterium tuberculosis* remains one of the world’s most devastating pathogens. For this reason, we developed a study involving 3D pharmacophore searching, selectivity analysis and database screening for a series of anti-tuberculosis compounds, associated with the protein kinases A, B, and G. This theoretical study is expected to shed some light onto some molecular aspects that could contribute to the knowledge of the molecular mechanics behind interactions of these compounds, with anti-tuberculosis activity. Using the Molecular Quantum Similarity field and reactivity descriptors supported in the Density Functional Theory, it was possible to measure the quantification of the steric and electrostatic effects through the Overlap and Coulomb quantitative convergence (alpha and beta) scales. In addition, an analysis of reactivity indices using global and local descriptors was developed, identifying the binding sites and selectivity on these anti-tuberculosis compounds in the active sites. Finally, the reported pharmacophores to PKn A, B and G, were used to carry out database screening, using a database with anti-tuberculosis drugs from the Kelly Chibale research group (http://www.kellychibaleresearch.uct.ac.za/), to find the compounds with affinity for the specific protein targets associated with PKn A, B and G. In this regard, this hybrid methodology (Molecular Mechanic/Quantum Chemistry) shows new insights into drug design that may be useful in the tuberculosis treatment today.

## 1. Introduction

One of the United Nations’ main focuses is to eradicate communicable diseases such as tuberculosis, which affect millions of people worldwide and causing more critical problems in countries with low- and middle-income. *Mycobacterium tuberculosis*, the causative agent of tuberculosis, is one of the most lethal human pathogens, further characterized by being strongly resistant to current treatments. Despite more than 100 years of research performed to date, the disease still kills about two million people every year around the world. According to the World Health Organization (WHO), a third of the world’s population carries the infection in an inactive form known as latency [1]. Our current inability to control the spread can be explained by the lack of an effective vaccine, multidrug-resistance [2,3,4] and the great adaptability of *Mycobacterium tuberculosis*, which has great capacity for mutation, in different environments, it [5,6,7,8,9,10].

In order to find new drug targets for tuberculosis treatment, in this study we analyzed the Protein Kinases (PKs) involved in tuberculosis. PKs, which are enzymes that catalyze the protein phosphorylation process, play an important role in controlling the proliferation and differentiation of eukaryotic cells in living organisms. One reason to investigate protein phosphorylation is that this rationalization represents an attractive drug target for a variety of diseases such as cancer [11], Alzheimer’s [12], chronic inflammations [13], etc. PKs present in the human body have been widely studied due to their use in therapeutic targets. However, not much is known about the PKs involved in tuberculosis. Therefore the binding sites associated to PKs A, B, and G of *Mycobacterium tuberculosis* are studied with a particular set of inhibitors to each PK.

The inhibitors used are a series of compounds of Pkn A reported by Sipos et al. [14], of Pkn B reported by Székely et al. [15], Loughheed et al. [16], Chapman et al. [17] and Naqvi et al. [18], finally of PKn G reported by Sipos et al. [14]. These ligands were used with the aim of obtaining new information about their stabilization in the active site.

The process of drug discovery is very complex and requires an interdisciplinary effort to design effective and commercially feasible drugs. In addition, the objective of drug design is to find a drug that can interact with a specific drug target and modify its activity. For this reason, we used a hybrid methodology to search new insights for tuberculosis treatment involving the application of Molecular Mechanics (MM) to protein treatment and consequently identifying the more active poses of the ligands involved in the anti-tuberculosis activity using computational techniques such as 3D pharmacophore searching and docking molecular [19,20,21] to each PK.

With the goal of studying the selectivity of these inhibitors in the active site, we used considerations of Quantum Chemistry (QC), specifically the Molecular Quantum Similarity (MQS) field [22,23,24,25] and chemical reactivity descriptors within the Density Functional Theory (DFT) framework [26]. In previous works, the present author has reported his approaches to relate Molecular Mechanics with Quantum Chemistry (MM/QM) [27]. Hopefully, this hybrid approach (MM/QM) provides new considerations about the interactions and selectivity of these ligands in the active sites of the PKs. Taking into account that selectivity is a very important aspect that is today widely studied in drug development with selective targets in diseases which are difficult to control like tuberculosis.

The final aspect of our work is to carry out a database screening using the 3D pharmacophores of PKn A, B and G reported on a database with anti-tuberculosis drugs, to find the compounds with affinity for the specific protein target associated with PKn A, B or G. To accomplish this we created a database using 183 anti-tuberculosis compounds reported by the Chibale group [28,29,30,31,32]. The compounds reported by Chibale are racemic mixtures. Taking this into account, the chiral isomers were characterized from the computational viewpoint to find the specific isomers interacting with each characterized pharmacophore.

## 2. Results

The outcomes in this work are distributed as follows: (i) 3D pharmacophore searching for the protein kinases A, B and G, (ii) analysis of the 3D pharmacophores using molecular quantum similarity and chemical reactivity descriptors (selectivity analysis), and (iii) 3D pharmacophore-based database screening.

### 2.1. 3D Pharmacophore Searching: Mechanic Molecular Approach

For the 3D pharmacophores analysis, we considered the classification given by Zuccotto’s group [33]. Zuccotto’s work explains the active kinase conformation through the “gatekeeper door”. In this sense, the compounds were classified as type I1/2 inhibitors; recognize the target kinases in the DFG “out” form for PKn A and DFG “in” for Pkn B, the Pkn G have DLG instead of DFG and is DLG “in”. While developing the docking analysis, hydrogen bonds on the hinge zone and the non-covalent interactions near the “gatekeeper door”, helix-αC, C-terminal and N-terminal, were taken into account. The non-covalent interaction involved backbone, side chain hydrogen bonding and aromatic-aromatic interactions. Ligands with high scores have combinations of these non-covalent interactions, while the ligands with lower scores have few to no interaction forces. Many of the top scoring ligands that form hydrogen bonds and aromatic-aromatic interactions with the amino acid residues, are close to the hinge zone.

The PKs A and B are transmembrane proteins, while the Pkn G is a cytosolic protein, therefore their active sites have different characteristics. The Pkn A and B consist of a transmembrane receptor with a tyrosine kinase domain, protruding into the cytoplasm. As for the Pkn G, the unique multidomain topology of Pkn G reveals a central kinase domain that is flanked by N- and C-terminal rubredoxin and tetratrico-peptide repeat domains. Directed mutagenesis suggests that the rubredoxin domain functions as a regulator of Pkn G kinase activity [34], which was taken with its respective ligand to develop the docking analysis. To generate the pharmacophores the hypotheses with highest scoring were chosen, its features are shown in Table 1.

**Figure 1:**
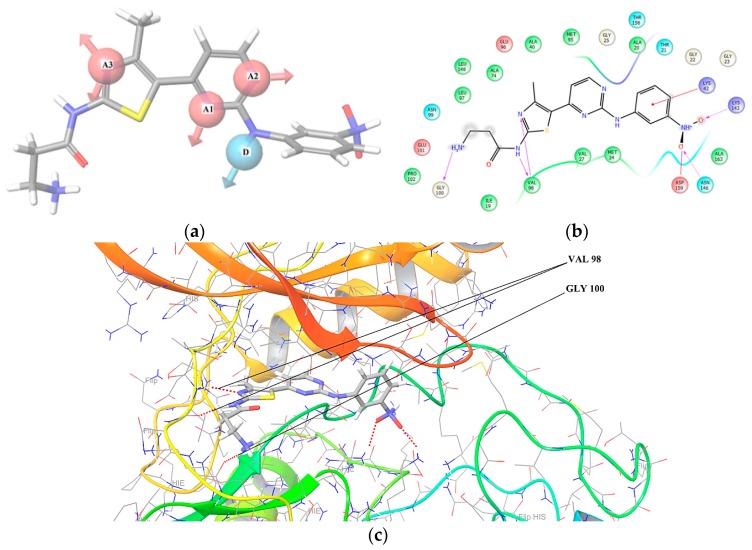
(**a**) Hypotheses AAAD to Pkn A on the Compound **1**, pIC_50_ = −1.569; (**b**) Ligands interactions between Gly 100, Val 98, Lys 42, 143, Asn 146 and Asp 159 residues and finally (**c**) Ligands interactions between Val 98 and Gly 100 residues on the Hinge zone to the compound **1**. PKn A (PBD code: 4OW8).

**Figure 2:**
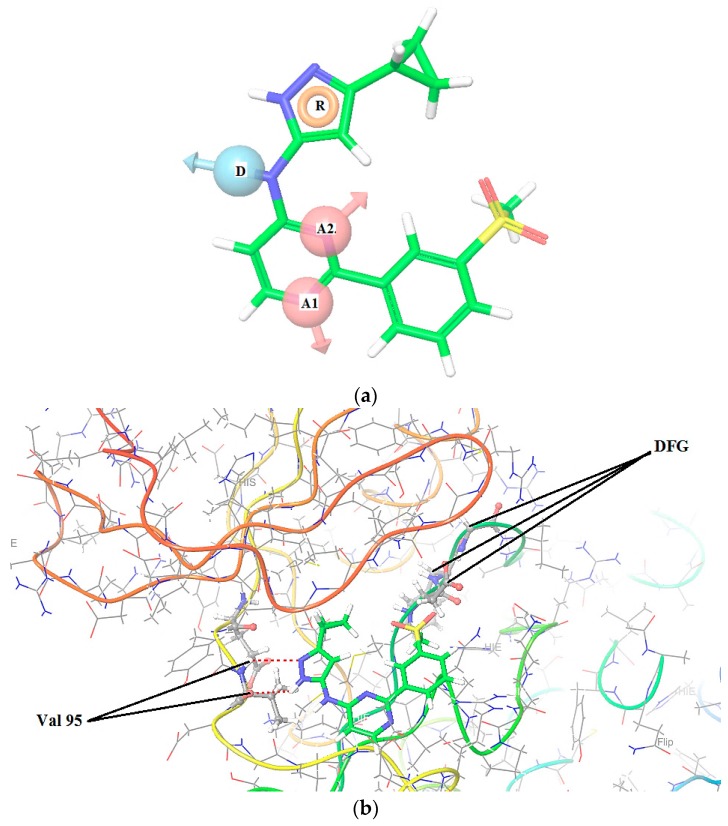
(**a**) Hypotheses AADR to Pk B on the Compound **7**, pIC_50_ = 1.066; (**b**) Zuccotto classification: type I1/2-Hinge Region/Back Pocket pharmacophore, DFG “in” Kinase (see Ref. [33]) and ligand interactions between Val 95 residues in the Hinge zone with the compound **7**. Pkn B (PBD code: 1O6Y).

**Figure 3:**
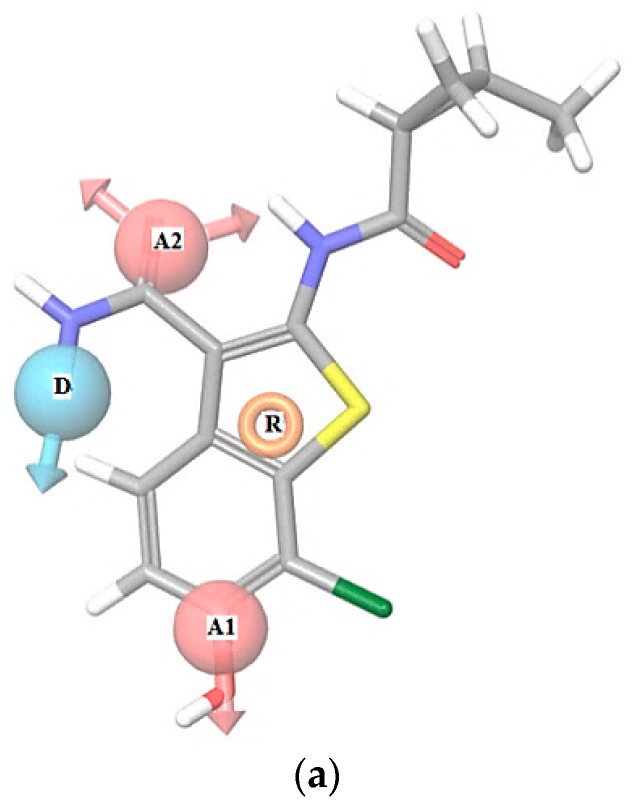

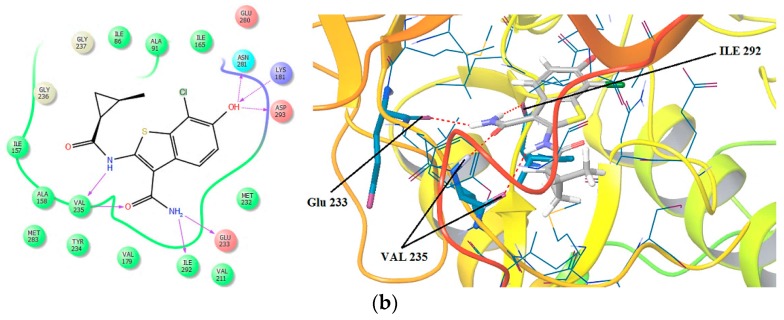
(**a**) Hypotheses AADR to Pk G on compound **23** (R isomer), pIC_50_ = 1.699; (**b**) Ligand interactions between Glu 233, Val 235 and Ile 292 residues in the hinge zone with compound **23**. PKn G (PBD code: 2PZI).

In Table 1 we can see a good reproducibility of each hypothesis shown above 70% to the molecular groups studied, these hypotheses are shown and characterized in Figure 1, Figure 2 and Figure 3.

In Figure 1, Figure 2 and Figure 3 are shown the interactions of the compounds **1** (Pkn A inhibitor), **7** (Pkn B inhibitor) and **23** (Pkn G inhibitor). Compound **1** presents three interactions on the hinge zone, while compound **7** presents two, and compound **23** also has three interactions. This criterion was crucial to define the actives poses and generate the hypothesis for each characterized pharmacophore. The results show the sites -H bond acceptor and -H bond donor on the hinge zone to all the ligands, establishing a structural model of the ligands on the active site with two or three interactions on this zone. With the focus to study the stabilization on the active site, we developed a study about chemical reactivity using DFT. The steric and electronic effects are characterized in terms of their chemical reactivity properties in the quantum chemistry context.

### 2.2. Selectivity Analysis: Quantum Chemistry Approach

#### 2.2.1. Molecular Quantum Similarity Study

To analyze the steric and electronic effects, we calculate the Carbó indices to and the overlap and Coulomb similarity, the Carbó indices are restring on the interval (0, 1] where 1 means self-similarity and 0 means null quantum similarity.

One election characteristic of the molecular groups, is that they must have the highest structural difference in order to generate 3D pharmacophores that may have the largest possible chemical information. This can be seen in Table S6, see Supporting Information (SI), where the majority of values corresponding to the structural similarity have low values, considering the fact that a good index of Carbó begins above 0.500. The highest comparison is between the compounds **2** and **3** with a value of 0.684 (see Table S6) and an Euclidean distance of 4.191 (Table S7, see SI). The lowest value is found in the comparison between the compounds **1** and **4**, with a value of 0.250 and an Euclidean distance of 6.317 (see Table S7, in the SI). Compound **1** has the highest activity (pIC_50_ = −1.568). We can see high structure differences along this molecular set. To study these features from the electronic point of view, the Coulomb indices are shown in Table S8, see SI.

**Figure 4:**
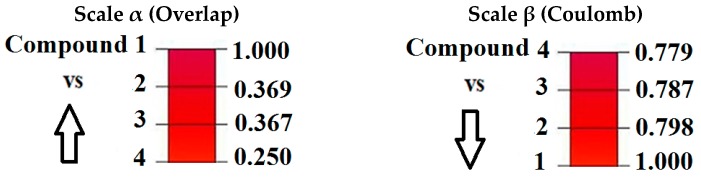
Convergence quantitative scales α to steric effects (overlap similarity) and β to electronic effects (coulomb similarity) for Pkn A inhibitors with respect to the most active compound **1**.

The highest value using the Coulomb index is between the compounds **3** and **4** (0.902), and an Euclidean distance of 29.140 (Table S9, in SI). However, the lowest value is between compounds **1** and **4** (0.779), and Euclidean distance of 40.699. To analyze the steric effects and electronic effects reported on the most active compound **1** we propose the convergence quantitative alpha (α) scales for steric effect and beta (β) for electronic effect (Figure 4), with the goal of studying the variability of the steric and electronic effects along the Pkn A inhibitors from the biological activity point of view.

In Figure 4 we can see the variability in the steric and electronic effects with the Carbó indices of the most active compound **1**. For this compound the highest similarity is with compound **2** (0.798) using the Coulomb operator (electronic effect) and with compound **3** (0.369) using the Dirac delta (steric effect).

**Figure 5:**
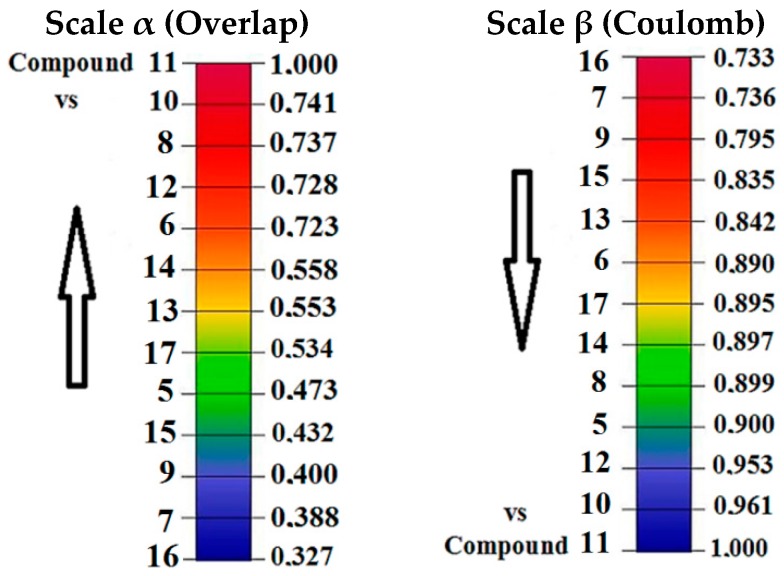
Convergence quantitative scales α for steric effects (overlap similarity) and β for electronic effects (Coulomb similarity) for Pkn B inhibitors with respect to the most active compound **11**.

For the Pkn B inhibitors, we can see the Carbó index in Tables S10–S13. For the overlap similarity, the highest comparison is between compounds **10** and **12** with a value of 0.832 (see Table S10) and Euclidean distance of 2.512 (see Table S11). The lowest value is obtained in the comparison between the compounds **10** and **16** with a value of 0.234 and Euclidean distance of 6.584 (see Table S11 in the SI). Among these Pkn B inhibitors, the compound with highest activity is **11** with pIC_50_ = 1.638, it has the highest structural difference with the compound **10** with a value of 0.741 (Table S10) and Euclidean distance of 3.241 (Table S11). The lowest structural difference for compound **7** is with compound **11** with a value of 0.388 and Euclidean distance of 4.825. To analyze the electronic effects, we calculated the Carbó indices (Table S12) using the Coulomb operators. The highest value in the Carbó index is obtain in the comparison between compounds **10** and **11** with the value 0.961 and Euclidean distance of 14.026 (see Table S13). The lowest value is obtained between the compounds **9** and **13** with a value of 0.589 and Euclidean distance of 43.709. To analyse the steric effects and electronic effects on the most active compound **11**, we can see the alpha (α) and beta (β) scales in Figure 5, to study the variability of the steric and electronic effects along the Pkn B inhibitors from the biological activity viewpoint.

In Figure 5 we can see the variability of the steric and electronic effects with the Carbó indices of the most active compound **11**. In both scales, compound **10** has the highest similarity and compound **16** has the lowest. These scales show how the chemical diversity selected can have influence on the biological activity, and also on the molecular space of the reported pharmacophore.

On the other hand, to the Pkn G inhibitors, the steric effects are shown in Table S14, see SI. In general, for this molecular group we obtained high values in the Carbó index of overlap; this fact is consistent with the common structural nucleus for this particular group. The highest value of overlap Similarity is obtained in the comparison between compounds **23** and **24** with the value of 0.993 and Euclidean distance of 0.492 (see Table S15), while the lowest value is obtained in the comparison between compounds **20** and **27** with a value of 0.532 and Euclidean distance of 4.556. These values show that the chemical diversity for this particular group is restricted with respect to the chemical diversity offered by the Pkn B inhibitors.

**Figure 6:**
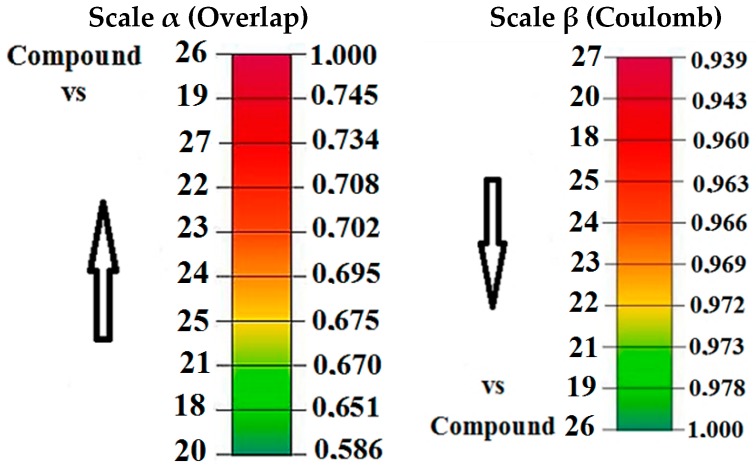
Convergence quantitative scales α for steric effects (overlap similarity) and β for electronic effects (coulomb similarity) for Pkn G inhibitors with respect to the most active compound **26**.

In general, the Coulomb indices show Carbó indices above of 0.900 (see Table S16). The highest value is obtained in the comparison between compounds **23** and **24** with a value of 0.999 and Euclidean distance of 1.246 (see Table S17, SI). The lowest value is obtained between compounds **24** and **27** with a value of 0.910 and Euclidean distance of 22.609. To analyze the variability of the steric and electronic effects on the biological activity, the alpha (α) and beta (β) scales are shown in Figure 6.

Figure 6 shows the variability of the steric and electronic effects on the biological activity of compound **26**, the most active in this molecular group. In both scales, compound **19** has the highest structural and electronic similarity with compound **26**.

On the other hand, in the analyses for the Pkn A, B and G inhibitors, the highest values were obtained using the Carbó Coulomb index. Taking into account these facts, we can say that the electronic effects have higher effect that the steric effects in the stabilization of these compounds in the active site associated to the Pkn A, B and G. With the aim to explore these outcomes a study on the chemical reactivity using global and local reactivity descriptors is developed in the DFT context.

#### 2.2.2. Chemistry Reactivity Study

To study the electronic effects reported by the Carbó indices in the previous section, we calculated the global reactivity indices such as chemical potential, hardness, softness, electrophilicity (see Table 2, Table 3 and Table 4), and local as the Fukui functions in order to analyze the stabilization and interactions of these compounds in the active site.

In Table 2, compound **1** has the lowest chemical potential (−4.124 eV), hardness (3.839 eV), softness (0.261 eV^−1^) and the highest electrophilicity (2.215 eV). This electrophilicity value can be related with the highest biological activity for this series, compound pIC_50_ = −1.568. Compound **1** shows the highest electronic and structural similarity when it is compared with compound **2** (0.787) and (0.369) (see Figure 4). Therefore, an electronic parameter such as the electrophilicity can be important in the stabilization of these anti-tuberculosis compounds in the active site. Table 3 shows the reactivity values to Pkn B inhibitors.

In Table 3, compound **12** has the highest chemical potential and hardness with a value of −2.769 eV and 5.888 eV. Consequently, with these values this compound has the lowest softness (S = 0.170 eV^−1^) and electrophilicity (ω = 0.651 eV). These values are consistent with the lowest biological activity of this compound (pIC_50_ = −1.200). On the other hand, the highest values of softness and electrophilicity correspond to compound **16**, S = 0.275 eV^−1^ and ω = 1.721 eV. This compound shows the highest steric and electronic effects when is compared with the most active compound **11**, with a Carbó index of overlap 0.327 and Coulomb index of 0.733 (see Figure 5), therefore the steric and electronic effects large can have influence in the stability of such compounds, in the active site and consequently these aspects show low biological activity from experimental point of view. To analyze the chemical reactivity associated to the Pkn G inhibitors, we can consider their global indices listed in Table 4.

In Table 4 we can see the values of electrophility (1.500 eV) and softness (0.229 eV^−1^) of compound **26**. These values are consistent with the highest biological activity to this compound (pIC_50_ = 2.000). However, the lowest values of electrophility and softness are found in compound **18** with 1.363 eV and 0.196 eV^−1^. Compound **18**’s values are related to the steric and electronic effects, quantified by the Carbó index of overlap 0.651 and Coulomb index of 0.960, with respect to the most active compound of this series compound **26** (Figure 6). Therefore, for this molecular group, the high biological activity may be related to the ability of the inhibitors, to receive electrons from the external environment. The stabilization can be determined by the retro-donor process on the hinge zone.

#### 2.2.3. Local Reactivity Study

**Figure 7:**
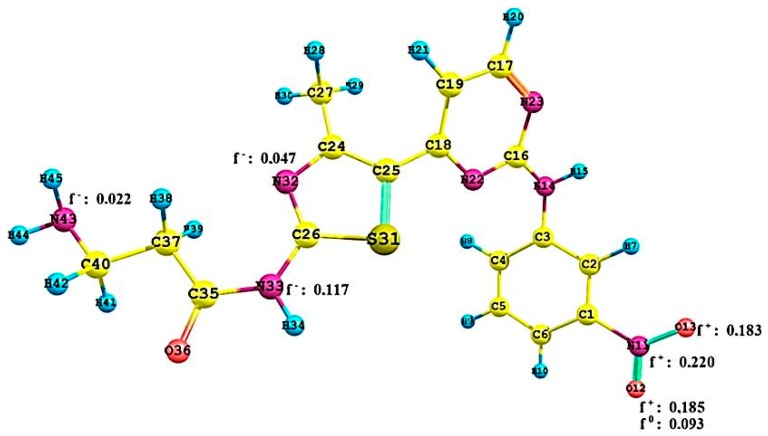
Local reactivity indices (Fukui functions) on the compound **1**, see Figure 1.

**Figure 8:**
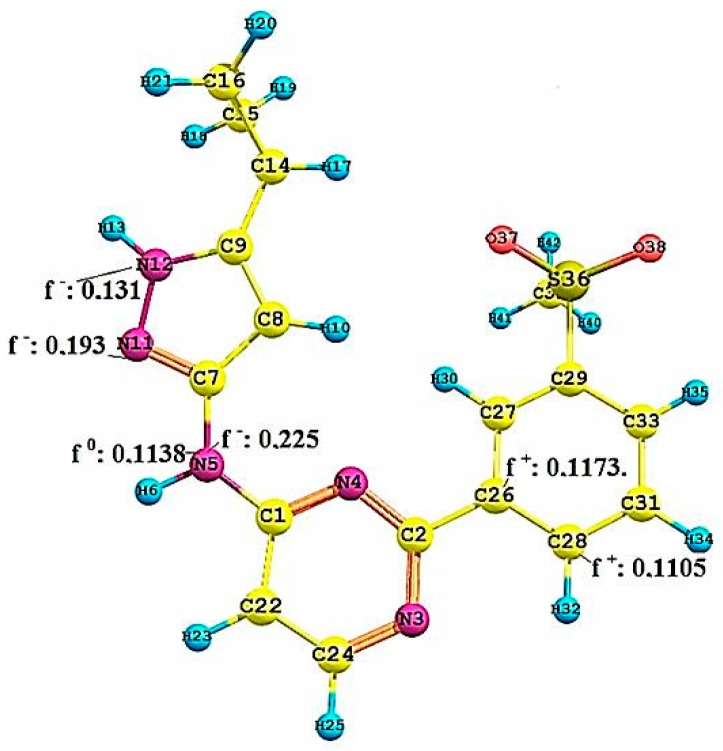
Local reactivity indices (Fukui functions) on the compound **7**, see Figure 2.

**Figure 9:**
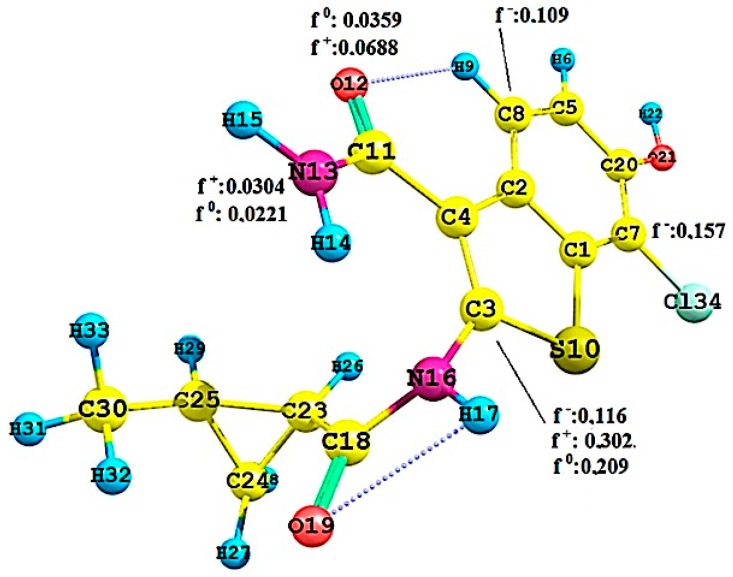
Local reactivity indices (Fukui functions) on the compound **23**, see Figure 3.

To quantify the molecular interactions and selectivity of the Pkn A, B and G inhibitors, we calculated the Fukui functions **f(r) ^−,+,0^** on the most active compounds **1** of Pkn A, **7** of Pkn B and **23** of Pkn G. The most significant values of these functions are shown in Figure 7, Figure 8 and Figure 9.

In Figure 7 we can see the interactions of N43 (f(r)^−^:0.022) with GLY 100, N33 (f(r)^−^:0.117) and N32 (f(r)^−^:0.047) with the VAL 98. The sites with affinity to nucleophilic attack are of the oxygen atoms (O12 (f(r)^+^:0.185) and O13 f(r)^+^:0.183) and on the nitrogen atom f(r)^+^:0.220. Finally, the site with neutral affinity is of the oxygen atom O12 (f(r)^0^:0.093) these interactions are responsible for the stabilization of the hinge zone and of the active site for these anti-tuberculosis compounds (see Figure 1).

Figure 8 shows the Fukui functions f(r)^−^ of the nitrogen atoms N12 (0.131), responsible for the hydrogen bond donor, and N11 (0.193) responsible for the acceptor site on the hinge zone. The other nitrogen atom, N5, besides showing a high f(r)^−^ = 0.225, shows a f(r)^0^ = 0.1138, representing that it can have either susceptibility to electrophilic attack, or toward neutral species. Therefore, this N5 can be crucial for the stabilization in the active site. The most sensitive values in the Fukui functions f(r)^+^ are shown on the carbon atoms C26 and C28, with values of 0.1173 and 0.1105. These values show the most susceptible zone to nucleophilic attacks. We can appreciate the nature of the molecular behaviour associated with the Pkn B inhibitors, and the type of stabilization that can happen on the active site. To know the nature of the chemical selectivity associated with the Pkn G inhibitors, is to know the Fukui functions of compound **23**.

Figure 9 shows the Fukui functions f(r)^+,0^ of the nitrogen atom N13, with values of 0.0304 and 0.0221. These values justify the interactions between the nitrogen atom N13 and the residues Ile 292 and Glu 233 on the hinge zone (one interaction of nucleophilic character and another of neutral nature). Another important interaction is between the oxygen atom O12 and the residue Val 235, which can be related to the value of f(r)^+^ = 0.0688.

The values of f(r)^−^ relevant are shown in the carbon atoms C8 and C3 with 0.109 and 0.116, representing the areas susceptible to electrophilic attacks. In general, these values show the Fukui functions and the molecular behaviour of these Pkn G inhibitors. Compound **23** can also have intramolecular hydrogen bonds, that may be important in the stabilization of the active site. We can see the nature of the selectivity and the interactions of Pkn G inhibitors and how they can interact with the residues on the hinge zone (see Figure 3).

### 2.3. 3D Pharmacophore-Based Database Screening on the Chibale’s Database (CD)

The database screening [35] is carried out using the Chibale database (see SI), with the pharmacophores reported in Figure 1, Figure 2 and Figure 3, to determine the compounds that can have affinity to each pharmacophores. The CD was created with 183 compounds [28,29,30,31,32] (see SI) and resulted in the following; 60 compounds have affinity with the Pkn A pharmacophore, 50 have affinity with the Pkn B, and other group of 30 compounds presented affinity with the Pkn G pharmacophore. These respectively affinities are shown in Table 5 and Table 7 through matching properties such as QPpolz: Predicted polarizability in Å^3^, SASA: Total Solvent-accessible Surface are in Å^2^, FOSA: Hydrophobic component of the SASA (saturated carbons and attached hydrogen), FISA: Hydrophilic component of the SASA (SASA on N, O and H on heteroatom), PISA: Pi (carbon and attached hydrogen) component of the SASA and finally WPS: Weakly polar component of the SASA (halogens, P and S).

In Table 5 and Table 6 we can see the 60 compounds matching the Pkn A pharmacophore. 50 compounds matching Pkn B, and 30 compounds with affinity to the Pkn G. In these tables there are also compounds with affinities to Pkn A, B and G simultaneously, compounds only with affinity to Pkn B and G, and finally compounds with only affinity to the Pkn A and B pharmacophores. The Tables predicted that polarizability values are into the recommended ranges for 95% of known drugs (13.0 to 70.0). On the other hand, the volume V_mol_ (the total volume of molecule enclosed by solvent-accessible molecular surface, in Å^3^ (probe radius 1.4 Å)) and the globularity descriptor Glob = (4πr^2^ )/Smol, where r is the radius of the sphere whose volume is equal to the molecular volume, are also in the recommended ranges (V_mol_: 500 to 2000 Å^3^) and (Glob: 0.75 to 0.95) [36,37,38,39,40,41,42,43,44].

In Table 7 we can see that the *R* and *S* isomers of compound **61** only present affinities to the pharmacophore Pkn G. Other important properties are the predicted skin permeability (Qlog K_p_) and the number of likely metabolic reactions (#metab). These properties also are in the recommended range (Qlog K_p_: −8.0 to −1.0) [45,46] and (#metab: 1 to 8) [36,37,38,39,40,41,42,43,44]. The results of this database screening may be helpful to characterize, from an experimental point of view, the *R* and *S* isomers, which is the goal of this work. We hope that this information can provide insights about the stereoisomers for a specific target Pkn A, B or G. To assess the ligands reported by the database screening, we used docking molecular with the structural model generated by the 3D pharmacophores, in order to verify the interaction of these ligands on the active site in each protein kinase.

## 3. Materials and Methods

The molecular dataset used in this study was taken from the literature as follow: four Pkn A inhibitors reported by Sipos et al. [14], 13 Pkn B inhibitor selected from the works of Székely et al. [15], Lougheed et al. [16], Chapman et al. [17] and Naqvi et al. [18] and 10 Pkn G inhibitors reported by Sipos et al. [14]). In this molecular dataset we considered structural diversity and uniform distribution of IC_50_. Logarithmic IC_50_ (μM) (pIC_50_ = −log IC_50_) was employed as a dependent variable instead of IC_50_. The pIC_50_ values of inhibition of the compounds are shown in Table 8 for Pkn A, Table 9 for Pkn B and Table 10 for Pkn G.

### 3.1. System Preparation to the Molecular Mechanic (MM) Approach

The crystal structure of PKn A (PBD code: 4OW8 [47]), B (PBD code: 1O6Y [48]) and PKn G (PBD code: 2PZI [34]) was prepared using Schrödinger Suite 2014-1’s Protein Preparation Wizard module [49,50], which refines the protein structure and optimizes the hydrogen bond (H-bond) network. Protonation states were determined using PropKa utility at a physiological pH. This was followed by a restrained molecular minimization using the Impact Refinement (Impref) module [51,52], with the heavy atoms restrained to remain within a root-mean-square deviation (RMSD) of 0.18 Å from the initial coordinates. The 3D molecular structures of the compounds were built using Maestro [53] and optimized using a B3LYP (hybrid-GGA exchange-correlation functional) at 6-311++G (2d,2p) level of theory [54,55] using Gaussian 09 [56], the ionization/tautomeric states were predicted at physiological pH conditions using Epik [57].

### 3.2. Docking Studies and Pharmacophore Research

Docking studies were carried out with Glide [58], using the Standard Precision (SP) mode with default parameters. Docking grid was generated with default settings centered at the co-crystallized ligand. A scaling factor down to 0.8 for the van der Waals radii of nonpolar protein atoms was used to accommodate the fact that the protein structure will not be optimized to fit larger ligands such as the studied in this work. The Induced Fit Docking (IFD) workflow [59,60] was employed to generate an alternative conformation of the receptor suitable to bind the studied ligands, by allowing the protein to undergo side-chain or backbone movements, or both, upon ligand docking. To develop the docking analysis we used protein-ligand complex molecular dynamics simulation of 25 ns in vacuo using GROMOS11 force field set, implemented through Gromacs 5.1.2 [61,62], to analyze the stability of each protein-ligand complex.

Finally, for the pharmacophore research we compared all the poses of the most and less active ligands for each congeneric family (PKn A, B and G), taking into account the extent of residue movement generated by the IFD calculation. The most energetically favourable conformation was selected by the best pose of each compound for further analysis, also ensuring that it exhibited interactions with the “hinge” residues of the PKs (i.e., Donor-Acceptor-Donor, DAD system). The pharmacophore research was carried out with Phase 3.7 [63] using four pharmacophore features: Hydrogen bond acceptor (A), Hydrogen bond donor (D), Hydrophobic group (H) and Aromatic ring (R); the pharmacophores of the best ligand poses in the active sites of the PKn A, B and G were examined and we chose the hypothesis with the highest score.

## 4. Theoretical and Computational Details to the Quantum Chemistry Approach

In this work, with the goal of studying the inhibitor from the quantum chemistry point of view, we used the MQS field and chemical descriptors within the DFT context, to analyze the group correlation and look for the facts that can determine the anti-tuberculosis activity of the Pkn A, B and G inhibitors considered.

### 4.1. Molecular Quantum Similarity: Steric and Electronic Effects Study

The similarity indices were introduced by Carbó and co-workers (see reviews on quantum similarity of [24,25,64,65,66]), and have been used to understand the steric and electronic effect on the molecular sets. The quantum similarity measure *Z_AB_* between compounds **A** and **B**, with electron density $\rho_{A}\left(\gamma_{1}\right)$ and $\rho_{B}\left(r_{2}\right)$ is defined, based on the idea of the minimizing of the expression for the Euclidean distance as:

(1) $$D_{AB}^{2}=\int^{\text{​}}{\left|\rho_{A}\left(r\right)-\rho_{B}\left(r\right)\right|}^{2}dr=\int {\left(\rho_{A}\left(r_{1}\right)\right)}^{2}\;dr_{1}+\int {\left(\rho_{B}\left(r_{2}\right)\right)}^{2}\;dr_{2}-2\int \rho_{A}\left(r_{1}\right)\left(r_{2}\right)dr_{1}dr_{2}=Z_{AA}+Z_{BB}-2Z_{AB}$$

Overlap integral involving the *Z_AB_* between the electronic density of the compound **A** and **B**, *Z_AA_* and *Z_BB_* are the self-similarity of compounds **A** and **B** [64,65,66]. The most common quantum similarity index is the one generalized by the cosine, introduced by Carbó et al. [64,65,66]. This index can be expressed mathematically as:

(2) $$I_{AB}=\frac{\int^{\text{​}}\rho_{A}\left(r_{1}\right)\rho_{B}\left(r_{2}\right)dr_{1}dr_{2}}{\sqrt{\int^{\text{​}}{\left(\rho_{A}\left(r_{1}\right)\right)}^{2}dr_{1}\int^{\text{​}}{\left(\rho_{B}\left(r_{2}\right)\right)}^{2}\;dr_{2}}}$$

or using the elements of *Z* in an operator (Ω):

(3) $$I_{AB}=\frac{Z_{AB}\left(Ω\right)}{\sqrt{Z_{AA}\left(Ω\right)Z_{BB}\left(Ω\right)}}$$

In Equation (3), the index is mathematically defined in the interval (0, 1) where 1 is self-similarity, and where only the measures of “shape similarity” are included. Another alternative is the Hodgkin-Richards index [67], which appears naturally when using the arithmetic mean and can be defined mathematically as:

(4) $$I_{AB}=\frac{2Z_{AB}\left(Ω\right)}{Z_{AA}\left(Ω\right)+Z_{BB}\left(Ω\right)}$$

This is (Ω) an operator for the measurement of quantum similarity. The Equation (4) shows another way to make Quantum Similarity Measures (QSM), but recent published work has shown that in fact, it is just an Euclidean distance, like shown in Equation (1) [68].

A simple way to make Quantum Similarity Measure (QSM) [22,23,24] involving two density functions, in the most usual form:

(5) $$Z_{AB}\left(Ω\right)=\iint \rho_{A}\left(r_{1}\right)Ω\left(r_{1},r_{2}\right)\rho_{B}\left(r_{2}\right)dr_{1}dr_{2}$$

In this equation $\rho_{A}\left(r_{1}\right)$ and $\rho_{B}\left(r_{2}\right)$ are the density functions to quantum objects ***A*** and ***B***, while $Ω\left(r_{1},r_{2}\right)$ is a positive define weight operator. When the operator is chosen as the Dirac Delta function: $\delta \left(r_{1}-r_{2}\right)$ we obtain overlap similarity measure, while Coulomb Similarity Measure appears when choosing: ${\left|r_{1}-r_{2}\right|}^{-1}$ These two operators are the most popular for similarity comparisons between molecules.

### 4.2. Molecular Alignment

To carry out the quantum similarity measures is an important optimal molecular alignment. As the integrals attached to the QSM produce, real positive defined results, the relative position problem can be addressed through a maximal QSM. For an overlap QSM, this situation can be expressed by means of the equation:

(6) $$\underset{T;\emptyset }{\max}Z_{AB}\left(T;\emptyset \right)=\underset{T;\emptyset }{\max}\int^{\text{​}}\rho_{A}\left(r\right)\rho_{B}\left(r|T;\emptyset \right)dr$$

In this equation, is implicitly supposed that $\rho_{B}\left(r\right)$ is translated and rotated by six possible ways, (T;φ) and are shown as explicit parameters in this integral [22,23,24,69]. This principle is used by the Topo Geometrical Superposition Algorithm (TGSA) [70] and is the program used in this study to calculate the Carbó indices using Equation (2).

### 4.3. Chemical Reactivity Descriptors: Selectivity Study

The global reactivity descriptors are defined within the DFT framework and were interpreted by Parr and coworkers [71,72,73,74,75,76,77,78]. The chemical potential can be written mathematically in terms of the energy of an electron in the frontier molecular orbitals (Higher Occupied Molecular Orbital) HOMO and (Lowest Unoccupied Molecular Orbital) LUMO as:

(7) $$\mu \approx \frac{\varepsilon_{L}+\varepsilon_{H}}{2}$$

From (8), one can obtain quantitative expression for the chemical hardness (*η*) [79,80,81,82,83], meaning that the opposition of the system to distort the electron cloud and mathematically can be written as:

(8) $$\eta \approx \varepsilon_{L}-\varepsilon_{H}$$

On the other hand, we have the global electrophilicity (ω) introduced by Parr et al. [84], which is a measure of the stabilization energy of the system when it is saturated by electrons from the external environment and represented mathematically as:

(9) $$\omega =\frac{\mu^{2}}{2\eta }$$

The Fukui function (f(r)) was one of the descriptors used in this work and defined as:

(10) $$f\left(r\right)={\left(\frac{\partial \rho \left(r\right)}{\partial N}\right)}_{V}={\left(\frac{\partial \mu }{\partial V\left(r\right)}\right)}_{N}$$

Due to that f(r) is discontinuous at integer values of 𝑁. There are three types according to Fukui et al., *f*^+^ which contain information on the reactivity local of nucleophilic attack, *f*^−^ (11) which does the same for an electrophilic attack. And finally *f*^0^, that measures the reactivity towards neutral or radical agents [84,85,86,87,88,89,90,91], using the condensation scheme on specific sites of the molecule to obtain the following Fukui indices [92,93,94,95]:

(11) $$f_{x}^{+}\left(r\right)\approx \rho_{N+1}^{x}\left(r\right)-\rho_{N}^{x}\left(r\right)\approx q_{x}\left(N+1\right)-q_{x}\left(N\right)\;f_{x}^{-}\left(r\right)\approx \rho_{N}^{x}\left(r\right)-\rho_{N-1}^{x}\left(r\right)\approx q_{x}\left(N\right)-q_{x}\left(N-1\right)\;f_{x}^{0}\left(r\right)\approx \left[\frac{f_{x}^{+}+f_{x}^{-}}{2}\right]\approx \frac{\rho_{N+1}^{x}\left(r\right)-\rho_{N-1}^{x}\left(r\right)}{2}\approx \frac{q_{x}\left(N+1\right)-q_{x}\left(N-1\right)}{2}$$

Hopefully using this equation scheme will help understanding the biological activity (selectivity) of the PKn A, B and G inhibitors studied from global and local point of view.

### 4.4. Creating the 3D Chibale’s Database: Database Screening

Computational Aided Drug Design (CADD) is presently a key component in the process of drug discovery and development associated with the tuberculosis disease. It offers great promise to drastically reduce cost and time requirements. In a pharmaceutical context, database screening is normally regarded as the top CADD tool to screen large libraries of chemical structures, and reduce them to a key set of likely drug candidates regarding a specific protein target [96,97].

In this work, the 3D Chibale database was created using 103 anti-tuberculosis compounds reported by the Chibale group [28,29,30,31,32]. Each anti-tuberculosis compound was characterized taking account their chiral centers (chiral isomers), which leaves the final molecular group with 183 compounds (see Tables S1–S5 in Supporting Information (SI)). This 3D database was created using the command line tools of Accompany Phase version 3.5, which is part of the Schrödinger Suite 2013 release [98,99,100]. It was used to evaluate the pharmacophores to Pkn A, B and G reported, to identify the compound that may have affinity with these specific protein targets.

## 5. Conclusions

In conclusion, the 3D pharmacophore reported were selected according to the hypotheses with highest score (Acceptor: A1/A2/A3/Donor:D) of Pkn A and (A/A/D/aromatic ring:R) of Pkn B and G. The three 3D pharmacophores generated were characterized using the molecular quantum similarity field and reactivity descriptors supported in the DFT framework. 

To develop the study of quantum similarity, we used the Carbó indices. Through these indices are proposed the convergence quantitative alpha (α) scale to steric effects and beta (β) to electronic effects with respect to the most active compound of each inhibitor set. In this analysis, the Carbó indices with the highest values are the Coulomb values. Considering that the electronic effects are more relevant than the steric, we develop a study using global reactivity descriptors such as Chemical Potential, Harness, Softness and the Fukui functions as local descriptor, to understand the interaction of these compounds in the active sites. 

The conclusion regarding the chemistry reactivity of Pkn A inhibitors is that the electronic factors as the electrophilicity can be important in the stabilization of these anti-tuberculosis compounds. In the active site of Pkn B inhibitors, there are steric and electronic effects that can have a big influence in the stability of such compounds in the active site. Consequently these aspects show low biological activity from experimental point of view. Regarding Pkn G inhibitors, the high biological activity may be related to the ability of the inhibitors to receive electrons from the external environment. The stabilization can be determined by the retro-donor process on the hinge zone. In this order of ideas, the reactivity descriptors reported can be related to the experimental data.

Finally, the database screening was developed using the Chibale’s Database created with 183 anti-tuberculosis compounds and using the Pkn A, B and G pharmacophores reported. We found 60 compounds with affinity to the Pkn A pharmacophore, 50 compounds for the PKn B and 30 compounds to the Pkn G. These predictions were tested with properties related such as **QPpolz**: Predicted Polarizability in Å^3^, **SASA**: total Solvent-Accessible Surface Are in Å^2^, **FOSA**: Hydrophobic component of the SASA (saturated carbons and attached hydrogen) and **WPS**: Weakly Polar Component of the SASA (halogens, P and S), among others. These properties related are within the recommended ranges for 95% of known drugs. In addition, the docking results for these ligands show the sites –H bond acceptor and –H bond donor, which are the characteristic interactions on the hinge zone in the active site, of each protein kinase studied.

## Figures and Tables

**Table 1:** Statistical data for the hypothesis of Pkn A, B and G, respectively.

PKn A	Survival	Site	Vector	Volume	Matches
AAAD *	3.145	0.85	0.956	0.484	4 of 4 compounds
ADRR	3.680	0.458	0.854	0.421	3 compounds
AADR	3.845	0.569	0.548	0.401	3 compounds
PKn B	Survival	Site	Vector	Volume	Matches
AADR *	4.316	0.93	0.992	0.627	9 of 13 compounds
AADR	4.308	0.91	0.988	0.640	7 compounds
DHRR	4.253	0.80	0.962	0.547	8 compounds
PKn G	Survival	Site	Vector	Volume	Matches
AADR *	3.056	0.47	0.888	0.696	10 of 10 compounds
AADR	2.466	0.46	0.753	0.257	10 compounds
AADR	2.180	0.16	0.710	0.311	10 compounds

* Hyphotheses selected.

**Table 2:** Global Chemical Reactivity Indices for Pkn A inhibitors. Chemical Potential (μ), Chemical Hardness (ƞ) and Electrophilicity (ω) in eV and Softness (S) in (eV)^−1^.

Compound	C. Potential (μ)	C. Hardness (η)	Softness (S)	Electrophilicity (ω)
**1**	−4.124	3.839	0.261	2.215
**2**	−3.485	3.543	0.282	1.714
**3**	−3.418	3.959	0.253	1.476
**4**	−3.491	3.154	0.317	1.932

**Table 3:** Global Chemical Reactivity Indices for Pkn B inhibitors. Chemical Potential (μ), Chemical Hardness (η) and Electrophilicity (ω) in eV and Softness (S) in (eV)^−1^.

Compound	C. Potential (μ)	C. Hardness (η)	Softness (S)	Electrophilicity (ω)
**5**	−3.575	5.655	0.177	1.129
**6**	−2.965	4.118	0.243	1.067
**7**	−3.575	4.089	0.245	1.563
**8**	−2.621	5.030	0.199	0.683
**9**	−2.981	4.351	0.230	1.021
**10**	−3.531	4.937	0.202	1.268
**11**	−3.313	3.764	0.266	1.458
**12**	−2.769	5.888	0.170	0.651
**13**	−2.887	4.630	0.216	0.900
**14**	−3.494	4.399	0.227	1.386
**15**	−3.111	4.705	0.213	1.028
**16**	−3.549	3.642	0.275	1.721
**17**	−3.252	3.428	0.292	1.543

**Table 4:** Global Chemical Reactivity Indices for Pkn G inhibitors. Chemical Potential (μ), Chemical Hardness (η) and Electrophilicity (ω) in eV and Softness (S) in (eV)^−1^.

Compound	C. Potential (μ)	C. Hardness (η)	Softness (S)	Electrophilicity (ω)
**18**	−3.730	5.103	0.196	1.363
**19**	−3.386	3.940	0.254	1.455
**20**	−3.905	5.127	0.195	1.487
**21**	−3.622	4.471	0.224	1.467
**22**	−3.731	4.473	0.224	1.556
**23**	−3.745	4.478	0.223	1.566
**24**	−4.131	4.651	0.215	1.835
**25**	−3.241	4.407	0.227	1.198
**26**	−3.623	4.375	0.229	1.500
**27**	−3.611	4.490	0.223	1.459

**Table 5:** Properties related and selected from the virtual screening on the Chibale’s Database (CB).

Compound with Pkn A Affinity	R	*n*	QPpolz ^d^	SASA ^e^	FOSA ^f^	FISA ^g^	PISA ^h^	WPSA ^i^	Clog P
**CB ^a^-11 ^b^ RR ^c^′**	H	3	33.435	519.287	246.394	108.866	134.095	29.932	2.12
**CB-11RS ′**	H	3	33.444	519.272	263.242	77.672	149.322	29.035	2.12
CB-11SR *	H	3	32.996	509.874	250.606	107.808	124.503	26.957	2.12
CB-11SS *	H	3	33.785	524.334	265.154	85.176	149.124	24.881	2.12
**CB-12RR ′**	Br	3	35.103	548.141	246.203	108.886	86.116	106.937	3.12
**CB-12RS ′**	Br	3	35.102	548.265	263.252	77.672	101.105	106.237	3.12
**CB-12SR ′**	Br	3	34.852	533.186	249.293	105.638	76.888	101.367	3.12
CB-12SS *	Br	3	35.515	554.864	265.193	85.184	102.419	102.068	3.12
**CB-13RR ′**	I	3	35.500	553.805	246.169	108.885	85.600	113.151	3.38
CB-13RS *	I	3	35.055	542.675	250.252	106.173	78.136	108.114	3.38
**CB-13SR ′**	I	3	35.498	553.944	263.253	77.671	100.562	112.458	3.38
CB-13SS *	I	3	35.911	560.530	265.180	85.178	101.877	108.294	3.38
**CB-14RR ′**	F	3	33.723	528.294	246.429	108.875	96.135	76.855	2.40
CB-14RS *	F	3	33.510	515.890	249.540	107.362	85.703	73.284	2.40
**CB-14SR ′**	F	3	33.727	528.286	263.248	77.672	111.462	75.905	2.40
CB-14SS *	F	3	34.140	534.883	265.189	85.187	85.187	71.739	2.40
**CB-15RR ′**	Cl	3	34.752	543.124	246.230	108.883	86.801	101.210	2.97
CB-15RS *	Cl	3	34.511	528.669	249.320	105.938	77.560	95.851	2.97
**CB-15SR ′**	Cl	3	34.752	543.237	263.253	77.672	101.828	100.483	2.97
CB-15SS *	Cl	3	35.165	549.828	265.186	85.186	103.137	96.319	2.97
**CB-16RR ′**	CH_3_	3	35.277	549.512	331.983	108.869	78.728	29.933	2.62
CB-16RS*	CH_3_	3	34.849	538.256	333.659	105.147	72.444	27.006	2.62
**CB-16SR ′**	CH_3_	3	35.306	551.436	351.239	77.670	93.498	29.029	2.62
CB-16SS*	CH_3_	3	35.715	557.934	353.127	85.178	112.768	24.885	2.62
**CB-17RR ′**	NO_2_	3	35.116	556.139	245.355	204.840	76.018	29.927	2.16
CB-17RS *	NO_2_	3	34.684	544.945	250.325	198.137	69.525	26.958	2.16
**CB-17SR ′**	NO_2_	3	35.157	557.839	263.310	174.709	90.792	29.029	2.16
**CB-17SS ′**	NO_2_	3	34.555	548.924	242.772	205.494	70.921	29.737	2.16
**CB-22RR ′**	H	5	36.703	547.504	292.222	101.786	124.211	29.285	3.24
CB-22RS*	H	5	37.311	561.538	269.138	131.258	151.763	9.379	3.24
**CB-22SR ′**	H	5	36.593	545.737	297.895	97.651	122.486	27.705	3.24
CB-22SS*	H	5	37.288	558.397	290.577	105.866	137.393	24.561	3.24
**CB-23RR ′**	Cl	5	38.013	571.319	292.026	101.818	76.893	100.582	4.09
**CB-23RS ′**	Cl	5	38.624	585.586	269.148	131.276	104.292	104.292	4.09
CB-23SR *	Cl	5	38.638	587.477	314.807	77.673	95.413	99.584	4.09
CB-23SS *	Cl	5	38.182	578.661	287.071	124.580	95.665	71.345	4.09
**CB-24RR ′**	F	5	36.985	556.516	292.243	101.807	86.274	76.192	3.52
**CB-24RS ′**	F	5	37.595	570.571	269.155	131.263	113.869	56.283	3.52
**CB-24SR ′**	F	5	37.691	574.185	314.788	77.664	106.723	75.009	3.52
CB-24SS *	F	5	37.061	548.024	313.187	78.434	97.486	58.918	3.52
**CB-25RR ′**	Br	5	38.384	576.756	291.976	101.823	101.823	106.298	4.24
CB-25RS *	Br	5	38.799	586.992	267.539	128.972	104.130	86.352	4.24
**CB-25SR ′**	Br	5	38.987	592.508	314.806	77.674	94.692	105.336	4.24
CB-25SS *	Br	5	38.522	583.254	287.106	124.245	94.983	76.920	4.24
**CB-26RR ′**	I	5	38.781	582.399	291.923	101.820	76.160	112.495	4.50
CB-26RS *	I	5	39.003	590.994	269.419	128.332	100.816	92.427	4.50
**CB-26SR ′**	I	5	39.384	598.197	314.810	77.679	94.151	111.557	4.50
CB-26SS *	I	5	39.003	582.006	283.206	121.666	94.068	83.066	4.50

**^a^ CB**: Chibale’s Database; **^b^** Numeration in the Chibale’s Database (see SI); **^c^** Chiral isomers: RR, RS, SR and SS; **^d^ QPpolz**: Predicted polarizability in Å^3^; **^e^ SASA**: Total Solvent-accessible Surface are in Å^2^; **^f^ FOSA**: Hydrophobic component of the SASA (saturated carbons and attached hydrogen); **^g^ FISA**: Hydrophilic component of the SASA (SASA on N, O and H on heteroatom); **^h^ PISA**: Pi (carbon and attached hydrogen) component of the SASA; **^i^ WPS**: Weakly polar component of the SASA (halogens, P and S); * Compound with Pkn A, B and G affinity; **′** Compound with only Pkn A and B affinity.

**Table 6:** The virtual screening on Chibale’s Database (CD).

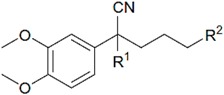		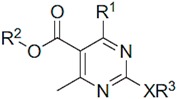		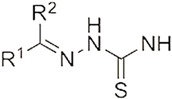	
**CB-61**		**CB-83-94**		**CB-90-98**	
**Compound with Pkn A Affinity**	**R^1^**	**R^2^**	**X**	**R^3^**	**pIC_50_**
**CB ^a^-61 ^b^ R ^c^ ′**	CH(CH_3_)_2_	1,2-Dimethoxy-phenylethylamine	-	-	−2.699
**CB-61S ′**	CH(CH_3_)_2_	1,2-Dimethoxy-phenylethylamine	-	-	−2.699
**CB-83**	H	C_2_H_5_	NH	H	-
**CB-84**	C_6_H_5_	C_2_H_5_	NH	H	-
**CB-85**	H	C_2_H_5_	NH	C_6_H_5_CH_2_	-
**CB-86**	C_6_H_5_	C_2_H_5_	NH	C_6_H_5_CH_2_	−1.805
**CB-88 ′**	C_6_H_5_	C_2_H_5_	NH	(CH_2_)_3_OH	>−2.107
**CB-90**	C_6_H_5_	C_2_H_5_	NH	n-C_4_H_9_	−2.071
**CB-92 ″**	C_6_H_5_	C_2_H_5_	NH	2-OHC_6_H_4_	−2.086
**CB-93 ′**	C_6_H_5_	C_2_H_5_	NH	4-OHC_6_H_4_	-
**CB-94 ″**	C_6_H_5_	C_2_H_5_	NH	3-NH_2_C_6_H_4_	−1.494
**CB-98 ′**	3-Methoxy-4-hydroxyphenyl	H	-	-	>−2.107

**^a^ CB**: Chibale’s Database; **^b^** Numbering in the Chibale Database (see SI); **^c^** Chiral isomerism; ′ Compound with Pkn A and G affinity; ″ Compound with Pkn A and B affinity.

**Table 7:** Properties related and selected from the virtual screening to the compounds of Table 6.

Compound	QPpolz ^d^	SASA ^e^	FOSA ^f^	FISA ^g^	PISA ^h^	WPSA ^i^
**CB ^a^-61 ^b^ R ^c^ ′**	46.733	809.665	554.715	61.543	193.408	0.000
**CB-61S ′**	46.917	824.012	572.502	56.988	194.522	0.000
**CB-83**	18.594	409.242	196.278	155.093	57.871	0.000
**CB-84**	27.711	506.534	191.029	126.786	188.719	0.000
**CB-85**	31.732	572.272	279.927	91.991	200.354	0.000
**CB-86**	41.980	693.239	280.021	62.464	350.753	0.000
**CB-88 ′**	34.639	641.288	314.312	123.633	203.343	0.000
**CB-90**	36.787	661.635	391.158	67.097	203.379	0.000
**CB-92 ″**	39.959	668.341	190.827	95.529	381.985	0.000
**CB-93 ′**	39.974	673.321	191.826	117.150	364.345	0.000
**CB-94 ″**	40.209	675.980	191.376	123.448	361.156	0.000
**CB-98 ′**	21.225	452.473	106.409	133.125	139.416	73.522

**^a^ CB**: Chibale’s Database; **^b^** Numbering in Chibale’s Database (see SI); **^c^** Chiral isomerism **^d^ QPpolz**: Predicted polarizability in Å^3^; **^e^ SASA**: Total Solvent-accessible Surface are in Å^2^; **^f^ FOSA**: Hydrophobic component of the SASA (saturated carbons and attached hydrogen); **^g^ FISA**: Hydrophilic component of the SASA (SASA on N, O and H on heteroatom); **^h^ PISA**: Pi (carbon and attached hydrogen) component of the SASA; **^i^ WPS**: Weakly polar component of the SASA (halogens, P and S); ′ Compound with Pkn A and G affinity; **″** Compound with Pkn A and B affinity.

**Table 8:** Structures, pIC_50_ values of the Pkn A inhibitors.

Compound	Structure	pIC_50_
**1**	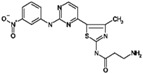	−1.569
**2**	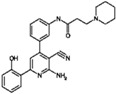	−1.839
**3**	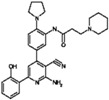	−1.875
**4**	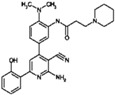	−1.934

**Table 9:** Structures, pIC_50_ values of the Pkn B inhibitors.

Compound	Structure	pIC_50_
**5**	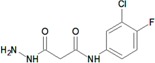	0.800
**6**	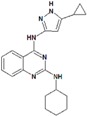	0.971
**7**	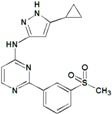	1.066
**8**	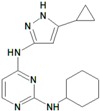	1.076
**9**	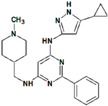	1.137
**10**	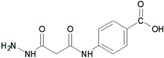	−0.360
**11**	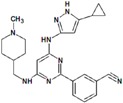	1.638
**12**	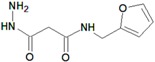	−1.200
**13**	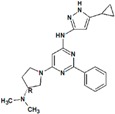	1.276
**14**	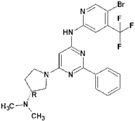	0.285
**15**	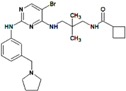	1.187
**16**	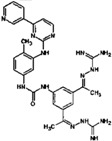	−0.375
**17**	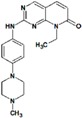	0.086

**Table 10:**
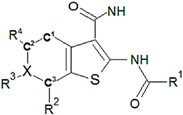
Structures, pIC_50_ values of the Pkn G inhibitors.

C ^a^	R^1^	R^2^	R^3^	R^4^	X	Bond C^3^-X	Bond C^1^-C^2^	pIC_50_
**18**	Cyclopropyl	H	H	H	C	C^3^-X	C^1^-C^2^	0.523
**19**	Cyclohexyl	H	-	H	O	C^3^-X	C^1^-C^2^	0.167
**20**	Cyclopropyl	H	-	H	O	C^3^-X	C^1^-C^2^	0.276
**21**	Isopropyl	H	–OH	H	C	C^3^=X	C^1^=C^2^	1.523
**22**	Cyclopropyl	Br	–OH	H	C	C^3^=X	C^1^=C^2^	1.699
**23**	Methylcyclopropyl	Cl	–OH	H	C	C^3^=X	C^1^=C^2^	1.699
**24**	Cyclopropyl	Cl	–OCH_3_	Cl	C	C^3^=X	C^1^=C^2^	1.398
**25**	Ethanol	H	–OH	H	C	C^3^=X	C^1^=C^2^	1.301
**26**	1,3-Benzodioxole	Cl	–OH	H	C	C^3^=X	C^1^=C^2^	2.000
**27**	Methylcyclopropyl	H	–OH	H	C	C^3^=X	C^3^=X	1.301

**^a^** C: compound.

## Supplementary Materials

The Supplementary Materials following are available online.
