# Supplementary material for: Molecular Quantum Similarity, Chemical Reactivity and Database Screening of 3D Pharmacophores of the Protein Kinases A, B and G from Mycobacterium tuberculosis
Source: Molecules. 2017 Jun 21;22(6):1027. doi: 10.3390/molecules22061027 (PMC6152632; doi:10.3390/molecules22061027)
Supplement: Supplementary file 1 [file molecules-22-01027-s001.pdf]

Molecular Quantum Similarity, Chemical Reactivity and Database Screening of 3D Pharmacophores of the Protein Kinases A, B and G from Mycobacterium Tuberculosis

Alejandro Morales-Bayuelo

Fondo Nacional de Desarrollo Científico y Tecnológico (FONDECYT), Proyecto Postdoctoral N⁰ 3150035, Talca, 3660300, Chile; alejandr.morales@uandresbello.edu

Supporting Information (SI)

Chibale's Database (DB):

Table S1. Chibale's Database. Tacked of the reference: *R. H. Hans; I. J. F. Wiid; P. D. van Helden; B. Wanc; S. G. Franzblau; J. Gut; P. J. Rosenthal;* ***K. Chibale****. Bioorg. Med. Chem. Lett.* ***2011****, 21, 2055–2058.*

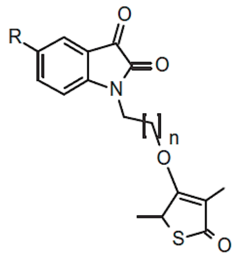

1-10

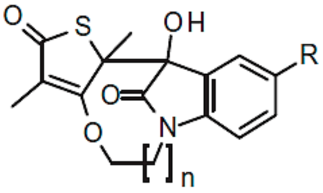

11-26

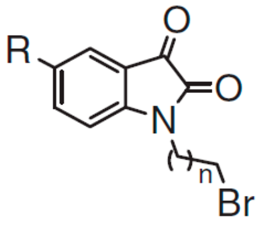

27-37

Compound	R	n	Clog P
**1R**	Br	1	2.88
**1S**	Br	1	2.88
**2R**	Cl	1	2.73
**2S**	Cl	1	2.73
**3R**	F	1	2.16
**3S**	F	1	2.16
**4R**	I	1	3.14
**4S**	I	1	3.14
**5R**	CH_3	1	2.34
**5S**	CH_3	1	2.34
**6R**	H	1	1.84
**6S**	H	1	1.84
**7R**	H	2	2.11
**7S**	H	2	2.11
**8R**	H	3	2.16
**8S**	H	3	2.16
**9R**	H	4	2.69
**9S**	H	4	2.69
**10R**	H	5	3.22
**10S**	H	5	3.22

**11RR**	H	3	2.12
**11RS**	H	3	2.12
**11SR**	H	3	2.12
**11SS**	H	3	2.12
**12RR**	Br	3	3.12
**12RS**	Br	3	3.12
**12SR**	Br	3	3.12
**12SS**	Br	3	3.12
**13RR**	I	3	3.38
**13RS**	I	3	3.38
**13SR**	I	3	3.38
**13SS**	I	3	3.38
**14RR**	F	3	2.40
**14RS**	F	3	2.40
**14SR**	F	3	2.40
**14SS**	F	3	2.40
**15RR**	Cl	3	2.97
**15RS**	Cl	3	2.97
**15SR**	Cl	3	2.97
**15SS**	Cl	3	2.97
**16RR**	CH_3	3	2.62
**16RS**	CH_3	3	2.62
**16SR**	CH_3	3	2.62
**16SS**	CH_3	3	2.62
**17RR**	NO_2	3	2.16
**17RS**	NO_2	3	2.16
**17SR**	NO_2	3	2.16
**17SS**	NO_2	3	2.16
**18RR**	H	4	2.68
**18RS**	H	4	2.68
**18SR**	H	4	2.68
**18SS**	H	4	2.68
**19RR**	Cl	4	3.53
**19RS**	Cl	4	3.53
**19SR**	Cl	4	3.53
**19SS**	Cl	4	3.53
**20RR**	Br	4	3.68
**20RS**	Br	4	3.68
**20SR**	Br	4	3.68
**20SS**	Br	4	3.68
**21RR**	I	4	3.94
**21RS**	I	4	3.94
**21SR**	I	4	3.94
**21SS**	I	4	3.94
**22RR**	H	5	3.24
**22RS**	H	5	3.24

**22SR**	H	5	3.24
**22SS**	H	5	3.24
**23RR**	Cl	5	4.09
**23RS**	Cl	5	4.09
**23SR**	Cl	5	4.09
**23SS**	Cl	5	4.09
**24RR**	F	5	3.52
**24RS**	F	5	3.52
**24SR**	F	5	3.52
**24SS**	F	5	3.52
**25RR**	Br	5	4.24
**25RS**	Br	5	4.24
**25SR**	Br	5	4.24
**25SS**	Br	5	4.24
**26RR**	I	5	4.50
**26RS**	I	5	4.50
**26SR**	I	5	4.50
**26SS**	I	5	4.50
**27**	H	1	1.30
**28**	H	2	1.62
**29**	H	3	2.00
**30**	I	3	3.31
**31**	Cl	3	2.90
**32**	Br	3	3.04
**33**	F	3	2.32
**34**	CH_3	3	2.50
**35**	NO_2	3	2.13
**36**	H	4	2.53
**37**	H	5	3.06

Table S2. Chibale's Database. Tacked of the reference: *R. H. Hans; E. M. Guantai; C. Lategan; P. J. Smith; B. Wanc; S. G. Franzblau; J. Gut; P. J. Rosenthal;* ***K. Chibale****. Bioorg. Med. Chem. Lett.* ***2010****, 20, 942–944.*

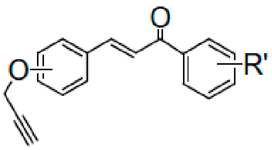

38-46

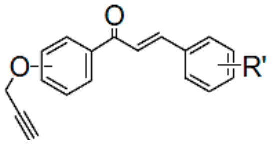

47-55

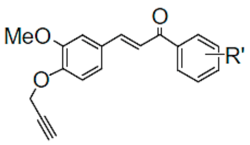

56-58

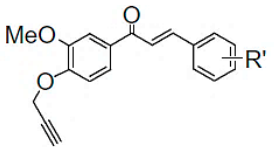

59-60

Compound	Substitution	R´	Clog P
**38**	Ortho	4-OMe	4.36
**39**	Ortho	2,4-diOMe	4.45
**40**	Ortho	2,3,4-triOMe	3.74
**41**	Meta	4-OMe	4.36
**42**	Meta	2,4-diOMe	4.45
**43**	Meta	2,3,4-triOMe	3.74
**44**	Para	4-OMe	4.36
**45**	Para	2,4-diOMe	4.45
**46**	Para	2,3,4-triOMe	3.74
**47**	Ortho	4-OMe	4.36
**48**	Ortho	2,4-diOMe	4.45
**49**	Ortho	2,3,4-triOMe	3.74
**50**	Meta	4-OMe	4.36
**51**	Meta	2,4-diOMe	4.45
**52**	Meta	2,3,4-triOMe	3.74
**53**	Para	4-OMe	4.36
**54**	Para	2,4-diOMe	4.45
**55**	Para	2,3,4-triOMe	3.74
**56**	-	4-OMe	4.10
**57**	-	2,4-diOMe	4.13
**58**	-	2,3,4-triOMe	3.38
**59**	-	2,4-diOMe	4.13
**60**	-	2,3,4-triOMe	3.38

Table S3. Chibale's Database. Tacked of the reference: *K. Singh; M. Kumar; E. Pavadai; K. N. Digby; F. Warner; P. G. Ruminski;* ***K. Chibale****. Bioorg. Med. Chem. Lett.* ***2014****, 24 2985–2990.*

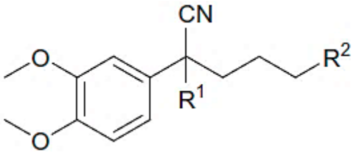

Compound	R^1	R^2	pIC_{99}
**61R**	$CH(CH_3)_2$	1,2-dymethoxy-phenyl-ethyl-amine	-2.699
**61S**	$CH(CH_3)_2$	1,2-dymethoxy-phenyl-ethyl-amine	-2.699
**62R**	$CH(CH_3)_2$	1,2-dymethoxy-phenyl-ethyl-methyl-amine	-2.699
**62S**	$CH(CH_3)_2$	1,2-dymethoxy-phenyl-ethyl-methyl-amine	-2.699
**63R**	$CH(CH_3)_2$	1,2-dymethoxy-phenyl-dyethyl-amine	-3.000
**63S**	$CH(CH_3)_2$	1,2-dymethoxy-phenyl-dyethyl-amine	-3.000
**64R**	$CH(CH_3)_2$	1,2-dymethoxy-phenyl-ethyl-propyl-amine	-2.699
**64S**	$CH(CH_3)_2$	1,2-dymethoxy-phenyl-ethyl-propyl-amine	-2.699
**65R**	$CH(CH_3)_2$	1,2-dymethoxy-phenyl-ethyl-methyl-phenyl-amine	-3.000
**65S**	$CH(CH_3)_2$	1,2-dymethoxy-phenyl-ethyl-methyl-phenyl-amine	-3.000
**66R**	$CH(CH_3)_2$	1,2-dymethoxy-phenyl-ethyl-cyclopropyl -amine	-2.097
**66S**	$CH(CH_3)_2$	1,2-dymethoxy-phenyl-ethyl-cyclopropyl -amine	-2.097
**67R**	$CH(CH_3)_2$	1,2-dymethoxy-phenyl-dymethyl -amine	>-3.000
**67S**	$CH(CH_3)_2$	1,2-dymethoxy-phenyl-dymethyl -amine	>-3.000
**68R**	$CH(CH_3)_2$	1,2-dymethoxy-phenyl-ethyl-methyl -amine	>-3.000
**68S**	$CH(CH_3)_2$	1,2-dymethoxy-phenyl-ethyl-methyl -amine	>-3.000
**69R**	$CH(CH_3)_2$	piperidine	-3.000
**69S**	$CH(CH_3)_2$	piperidine	-3.000
**70R**	$CH(CH_3)_2$	Oxy-piperidine	-3.000

**70S**	$CH(CH_3)_2$	Oxy-piperidine	−3.000
**71R**	$CH(CH_3)_2$	Hydroxy-piperidine	-3.000
**71S**	$CH(CH_3)_2$	Hydroxy-piperidine	-3.000
**72R**	$CH(CH_3)_2$	dypiperidine	-2.699
**72S**	$CH(CH_3)_2$	dypiperidine	-2.699
**73R**	$CH(CH_3)_2$	Hydroxy-phenyl-piperidine	-2.398
**73S**	$CH(CH_3)_2$	Hydroxy-phenyl-piperidine	-2.398
**74R**	$CH(CH_3)_2$	phenyl-piperazine	-2.398
**74S**	$CH(CH_3)_2$	phenyl-piperazine	-2.398
**75R**	$CH(CH_3)_2$	2-piridine- piperazine	-2.398
**75S**	$CH(CH_3)_2$	2-piridine- piperazine	-2.398
**76R**	$CH(CH_3)_2$	2-Fluore-phenyl-piperazine	-1.795
**76S**	$CH(CH_3)_2$	2-Fluore-phenyl-piperazine	-1.795
**77R**	H	1,2-dymethoxy-phenyl-ethyl-methyl-amine	-3.000
**77S**	H	1,2-dymethoxy-phenyl-ethyl-methyl-amine	-3.000
**78R**	CH_3	1,2-dymethoxy-phenyl-ethyl-methyl-amine	-3.000
**78S**	CH_3	1,2-dymethoxy-phenyl-ethyl-methyl-amine	-3.000
**79R**	$CH(CH_3)_2$	1,2-dymethoxy-phenyl-ethyl-methyl-amine	-3.000
**79S**	$CH(CH_3)_2$	1,2-dymethoxy-phenyl-ethyl-methyl-amine	-3.000
**80R**	$(CH_2)_2CH_3$	1,2-dymethoxy-phenyl-ethyl-methyl-amine	-2.699
**80S**	$(CH_2)_2CH_3$	1,2-dymethoxy-phenyl-ethyl-methyl-amine	-2.699
**81R**	Cyclopentyl	1,2-dymethoxy-phenyl-ethyl-methyl-amine	-2.097
**81S**	Cyclopentyl	1,2-dymethoxy-phenyl-ethyl-methyl-amine	-2.097
**82R**	Ciclohexyl	1,2-dymethoxy-phenyl-ethyl-methyl-amine	-1.796
**82S**	Ciclohexyl	1,2-dymethoxy-phenyl-ethyl-methyl-amine	-1.796

Table S4. Chibale's Database. Tacked of the reference: *K. Singh; K. Singh; B. Wan; S. Franzblau;* ***K. Chibale****; J. Balzarini. Eur. J. Med. Chem.* ***2011****, 46, 2290-2294.*

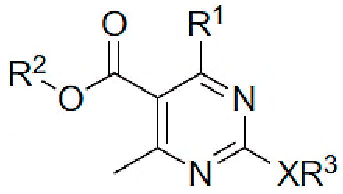

Compound	R^1	R^2	X	R^3	pIC_{50}
**83**	H	C_2H_5	NH	H	-
**84**	C_6H_5	C_2H_5	NH	H	-
**85**	H	C_2H_5	NH	C_6H_5 CH_2	-
**86**	C_6H_5	C_2H_5	NH	C_6H_5 CH_2	-1.805
**87**	C_6H_5	CH_3	NH	C_6H_5 CH_2	>-2.107
**88**	C_6H_5	C_2H_5	NH	$(CH_2)_3OH$	>-2.107
**89**	C_6H_5	C_2H_5	NH	$CH(CH_3)_2$	
**90**	C_6H_5	C_2H_5	NH	N-C_4H_9	-2.071
**91**	C_6H_5	C_2H_5	NH	CH_2CH_2 (1H-indol-2-yl)	-
**92**	C_6H_5	C_2H_5	NH	2-OHC_6H_4	-2.086
**93**	C_6H_5	C_2H_5	NH	4-OHC_6H_4	-
**94**	C_6H_5	C_2H_5	NH	3-$NH_2C_6H_4$	-1.494
**95**	C_6H_5	C_2H_5	NH	-$(CH_2)_2$-CH_2-(CH_2)-	>-2.107
**96**	C_6H_5	C_2H_5	N	-$(CH_2)_2$-O-$(CH_2)_2$-	-2.098
**97**	C_6H_5	C_2H_5	O	C_2H_5	-

Table S5. Chibale's Database. Tacked of the reference: *S. D. Khanye; B. Wan; S. G. Franzblau; J. Gut; P. J. Rosenthal; G. S. Smith;* ***K. Chibale****. J. Organomet. Chem.* ***2011****, 696, 3392-3396.*

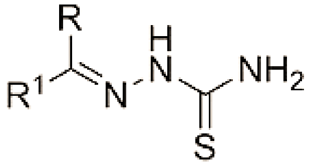

Compound	R	R^1	pIC_{50}
**98**	H	3-methoxy-4-hydroxy-Fenyl	>-2.107
**99**	CH_3	3, 4-dicloro-fenyl	-1.068
**100**	CH_3	3-bromo-fenyl	>-2.107
**101**	CH_3CH_2	3-bromo-fenyl	-2.107
**102**	CH_3CH_2	3, 4-dicloro-fenyl	>-2.107
**103**	CH_3	4-bromo-fenyl	>-2.107

Molecular Quantum Similarity Indices.

Table S6. Carbó index of Overlap for Pkn A inhibitors (see **Table 8**).

C, OI[a]	1	2	3	4
1	1.000			
2	0.369	1.000		
3	0.367	0.684	1.000	
4	0.250	0.409	0.441	1.000

[a]C: Compound, OI: Overlap Index.

Table S7. Euclidean Distance of Overlap for Pkn A inhibitors.

C, ED[a]	1	2	3	4
1	0.000			
2	5.643	0.000		
3	5.874	4.191	0.000	
4	6.317	5.663	5.699	0.000

[a]**C**: Compound, **ED**: Euclidean Distance.

Table S8. Carbó index of Coulomb for Pkn A inhibitors (see **Table 8**).

C, CI[a]	1	2	3	4
1	1.000			
2	0.798	1.000		
3	0.787	0.893	1.000	
4	0.779	0.855	0.902	1.000

[a]C: Compound, CI: Coulomb Index.

Table S9. Euclidean Distance of Coulomb for Pkn A inhibitors.

C, ED[a]	1	2	3	4
1	0.000			
2	36.434	0.000		
3	41.062	30.021	0.000	
4	40.699	33.932	29.140	0.000

[a]**C**: Compound, **ED**: Euclidean Distance.

Table S10. Carbó index of Overlap for Pkn B inhibitors (see **Table 9**).

C, OI[a]	5	6	7	8	9	10	11	12	13	14	15	16	17
5	1.000												
6	0.268	1.000											
7	0.440	0.365	1.000										
8	0.266	0.792	0.287	1.000									
9	0.468	0.291	0.634	0.298	1.000								
10	0.438	0.654	0.390	0.720	0.308	1.000							
11	0.473	0.723	0.388	0.737	0.400	0.741	1.000						
12	0.481	0.644	0.444	0.581	0.397	0.832	0.728	1.000					

13	0.289	0.576	0.443	0.634	0.289	0.592	0.553	0.419	1.000				
14	0.237	0.694	0.394	0.627	0.244	0.572	0.558	0.549	0.520	1.000			
15	0.364	0.418	0.406	0.410	0.336	0.429	0.432	0.424	0.368	0.463	1.000		
16	0.460	0.327	0.390	0.336	0.290	0.234	0.327	0.423	0.463	0.315	0.330	1.000	
17	0.458	0.462	0.509	0.454	0.323	0.540	0.534	0.615	0.498	0.412	0.448	0.318	1.000

Table S11. Euclidean Distance of Overlap for Pkn B inhibitors.

C, ED[a]	5	6	7	8	9	10	11	12	13	14	15	16	17
5	0.000												
6	5.350	0.000											
7	4.363	5.026	0.000										
8	5.508	3.122	5.464	0.000									
9	4.070	5.123	3.419	5.251	0.000								
10	4.536	3.824	4.763	3.536	4.877	0.000							
11	4.450	3.452	4.825	3.455	4.612	3.241	0.000						
12	4.208	3.786	4.396	4.223	4.389	2.512	3.236	0.000					
13	5.275	4.346	4.708	4.131	5.129	4.151	4.384	4.820	0.000				
14	6.046	4.073	5.458	4.554	5.891	4.720	4.818	4.762	5.074	0.000			
15	5.246	5.305	5.104	5.453	5.232	5.130	5.163	5.037	5.529	5.518	0.000		
16	5.497	6.310	5.823	6.366	6.099	6.584	6.239	5.684	5.665	6.762	6.467	0.000	
17	4.573	4.860	4.384	5.015	4.961	4.367	4.443	3.895	4.693	5.571	5.134	6.317	0.000

[a]**C**: Compound, **ED**: Euclidean Distance.

Table S12. Carbó index of Coulomb for Pkn B inhibitors (see **Table 9**).

C, CI[a]	5	6	7	8	9	10	11	12	13	14	15	16	17
5	1.000												
6	0.777	1.000											
7	0.877	0.666	1.000										
8	0.778	0.965	0.716	1.000									
9	0.846	0.655	0.887	0.675	1.000								
10	0.853	0.910	0.776	0.925	0.774	1.000							
11	0.900	0.890	0.736	0.899	0.795	0.961	1.000						
12	0.887	0.875	0.808	0.863	0.789	0.969	0.953	1.000					
13	0.774	0.897	0.841	0.928	0.589	0.932	0.842	0.701	1.000				
14	0.780	0.970	0.831	0.947	0.763	0.925	0.897	0.896	0.874	1.000			
15	0.794	0.836	0.830	0.828	0.759	0.843	0.835	0.820	0.796	0.874	1.000		
16	0.805	0.683	0.799	0.683	0.650	0.698	0.733	0.769	0.853	0.759	0.787	1.000	
17	0.870	0.807	0.854	0.821	0.714	0.883	0.895	0.915	0.815	0.788	0.792	0.794	1.000

[a]C: Compound, CI: Coulomb Index.

Table S13. Euclidean Distance of Coulomb for Pkn B inhibitors.

C, ED[a]	5	6	7	8	9	10	11	12	13	14	15	16	17
5	0.000												
6	34.606	0.000											
7	18.446	40.538	0.000										
8	36.673	14.941	39.947	0.000									
9	19.719	41.11	17.222	42.397	0.000								
10	27.279	22.507	32.025	21.809	32.610	0.000							

11	22.591	24.722	33.275	24.977	30.302	14.026	0.000						
12	21.295	26.322	26.813	28.981	28.085	13.047	14.921	0.000					
13	34.609	24.620	30.367	21.269	43.709	19.559	29.395	39.319	0.000				
14	39.249	15.284	35.985	19.339	41.100	23.487	27.168	28.240	29.438	0.000			
15	39.427	34.015	37.069	35.127	42.358	33.220	34.002	35.626	37.719	30.667	0.000		
16	48.184	53.255	48.202	53.458	56.833	51.996	49.466	47.505	38.722	47.559	44.986	0.000	
17	26.887	33.097	27.878	32.914	36.500	24.881	23.262	20.993	32.264	37.488	37.796	44.481	0.000

[a]**C**: Compound, **ED**: Euclidean Distance.

Table S14. Carbó index of Overlap for Pkn G inhibitors (see **Table 10**).

C, OI[a]	18	19	20	21	22	23	24	25	26	27
18	1.000									
19	0.685	1.000								
20	0.734	0.775	1.000							
21	0.800	0.872	0.868	1.000						
22	0.688	0.894	0.814	0.800	1.000					
23	0.609	0.850	0.739	0.767	0.904	1.000				
24	0.634	0.853	0.730	0.755	0.892	0.993	1.000			
25	0.631	0.884	0.741	0.801	0.885	0.915	0.911	1.000		
26	0.651	0.745	0.586	0.670	0.708	0.702	0.695	0.675	1.000	
27	0.640	0.661	0.532	0.582	0.620	0.616	0.613	0.623	0.734	1.000

[a]C: Compound, OI: Overlap Index.

Table S15. Euclidean distances of Overlap for Pkn G inhibitors.

C, ED[a]	18	19	20	21	22	23	24	25	26	27
18	0.000									
19	3.216	0.000								
20	3.078	2.891	0.000							
21	2.518	2.077	2.203	0.000						
22	3.239	1.940	2.645	2.622	0.000					
23	3.613	2.297	3.131	2.824	1.854	0.000				
24	3.508	2.279	3.189	2.903	1.961	0.492	0.000			
25	3.558	2.043	3.149	2.649	2.057	1.762	1.810	0.000		
26	3.492	3.057	4.001	3.428	3.297	3.323	3.369	3.504	0.000	
27	3.873	3.819	4.556	4.183	4.058	4.068	4.089	4.063	3.441	0.000

[a]**C**: Compound, **ED**: Euclidean Distance.

Table S16. Carbó index of Coulomb for Pkn G inhibitors (see **Table 10**).

C, CI[a]	18	19	20	21	22	23	24	25	26	27
18	1.000									
19	0.963	1.000								
20	0.941	0.957	1.000							
21	0.981	0.987	0.964	1.000						
22	0.951	0.980	0.971	0.977	1.000					
23	0.935	0.971	0.951	0.972	0.988	1.000				
24	0.941	0.972	0.950	0.971	0.987	0.999	1.000			

**25**	**0.942**	**0.977**	**0.939**	**0.970**	**0.977**	**0.984**	**0.983**	**1.000**		
**26**	**0.960**	**0.978**	**0.943**	**0.973**	**0.972**	**0.969**	**0.966**	**0.963**	**1.000**	
**27**	**0.946**	**0.938**	**0.911**	**0.922**	**0.920**	**0.912**	**0.910**	**0.918**	**0.939**	**1.000**

[a]C: Compound, CI: Coulomb Index.

Table S17. Euclidean distances of Coulomb for Pkn G inhibitors.

**C, ED[a]**	**18**	**19**	**20**	**21**	**22**	**23**	**24**	**25**	**26**	**27**
**18**	**0.000**									
**19**	**11.270**	**0.000**								
**20**	**16.237**	**13.729**	**0.000**							
**21**	**7.867**	**6.810**	**13.404**	**0.000**						
**22**	**13.274**	**8.338**	**11.399**	**9.363**	**0.000**					
**23**	**15.600**	**10.460**	**14.404**	**10.659**	**6.758**	**0.000**				
**24**	**14.907**	**10.374**	**14.623**	**10.946**	**7.177**	**1.246**	**0.000**			
**25**	**15.660**	**10.067**	**16.114**	**11.885**	**9.995**	**8.260**	**8.432**	**0.000**		
**26**	**12.266**	**8.926**	**15.551**	**10.164**	**10.173**	**10.879**	**11.158**	**12.248**	**0.000**	
**27**	**20.063**	**20.148**	**22.069**	**22.594**	**21.746**	**22.381**	**22.609**	**21.446**	**19.593**	**0.000**

[a]**C**: Compound, **ED**: Euclidean Distance.
